# Super-efficient drilling of metals with ultrafast non diffractive laser beams

**DOI:** 10.1038/s41598-022-05967-5

**Published:** 2022-02-08

**Authors:** Huu Dat Nguyen, Enrique Moreno, Anton Rudenko, Nicolas Faure, Xxx Sedao, Cyril Mauclair, Jean-Philippe Colombier, Razvan Stoian

**Affiliations:** 1 Laboratoire Hubert Curien, UMR 5516 CNRS, Université Jean Monnet, 42000 Saint-Étienne, France; 2 GIE Manutech-USD, 42000 Saint-Étienne, France

**Keywords:** Laser material processing, Ultrafast lasers

## Abstract

A highly efficient drilling process is found in non-transparent metallic materials enabled by the use of non-diffractive ultrafast Bessel beams. Applied for deep drilling through a 200 μm-thick steel plate, the Bessel beam demonstrates twofold higher drilling efficiency compared to a Gaussian beam of similar fluence and spot size. Notwithstanding that surface ablation occurs with the same efficiency for both beams, the drilling booster results from a self-replication and reconstruction of the beam along the axis, driven by internal reflections within the crater at quasi-grazing incidence, bypassing potential obstacles. The mechanism is the consequence of an oblique wavevectors geometry with low angular dispersion and generates a propagation length beyond the projection range allowed by the geometry of the channel. With only the main lobe being selected by the channel entrance, side-wall reflection determines the refolding of the lobe on the axis, enhancing and replicating the beam multiple times inside the channel. The process is critically assisted by the reduction of particle shielding enabled by the intrinsic self-healing of the Bessel beam. Thus the drilling process is sustained in a way which is uniquely different from that of the conventional Gaussian beam, the latter being damped within its Rayleigh range. These mechanisms are supported and quantified by Finite Difference Time Domain calculations of the beam propagation. The results show key advantages for the quest towards efficient laser drilling and fabrication processes.

## Introduction

Ultrafast pulsed lasers have been recognized for several decades as precision tools for micro-processing of materials. Their employment, for example, for structuring of metals^1,2^ follows already a robust path to industrial integration. The technique takes advantage of the ultrafast deposition of energy into the material under non-equilibrium conditions promoting swift ablation and resulting in a significant reduction of the collateral damage to the surrounding environment^3–5^. Implicitly related to the energy confinement and reduced diffusion, ultrafast laser ablation suffers nevertheless of a relatively low yield^6,7^, defined by an energy deposition length restricted between an optical and a characteristic thermal penetration depth during the process duration^8^. The nature and speed of energy transport is critical to ensure the necessary energy density for ablation. Alongside precision that sets the quality of the process, the processing yield is therefore a key process parameter, being related to the speed of evacuating material from the processed area and the time-effectiveness of structuring. The efficiency of laser ablation of metallic materials has been evaluated against several processing variables, i.e., fluence^1,8–11^, pulse duration^12–15^, repetition rate^16–21^, and processing wavelength^22^. These strategies aim to optimize the photon cost of the process and the energy penetration depth for a relatively efficient material removal. The recent strategies based on burst modes with pulse frequencies in the MHz, GHz, or THz follow an approach of optimizing the physical process of energy deposition, synchronizing it with either the thermal transport or with the hydrodynamic flow and the evacuation rate^23–29^. Confining deposition volumes and channeling the energy preferentially to the ablation products can determine an optimal balance between yield, precision, and the nature of the ejecta^30^. The objective is an overall optimized process that combines energy feedthrough, precision, and processing speed, particularly important for deep drilling applications^31^.

An alternative strategy which has an important influence in improving material processing relies on the design of the laser beam shape in radial and axial directions. While the Gaussian profile is the most commonly used beam shape, its intrinsic properties during interaction with matter, i.e., non-linear distortions in high intensity regimes and low aspect ratios of the focal volume, generate limitations for processing and, particularly, for deep-drilling of taper-free channels^32–34^. Same limitations apply for beam in-plane shaping, where particular forms can be acquired in an imaging plane and then projected onto the sample^35^. As these are consequences of diffraction, using a diffraction-free beam configuration maximizing the focal volume could offer significant advantages for overcoming the conventional Gaussian limitation and further advancing the laser processing. Bessel beams^36^ represent a class of non-diffractive optical fields that ideally do not spread over a long propagation distance^37^. Their formation is determined by conical interference of wavefronts upon propagation through a conical lens, which produces an invariant intensity profile in the axial direction along with a self-reconstruction property. As all the wavefronts arrive at the same angle to the propagation axis, the Bessel beam is considered to be free from spherical aberrations when crossing interfaces. In addition, the intensity of the beam remains modest before reaching the central core region, which makes it less affected by nonlinear effects, i.e., nonlinear distortion and self-focusing^38^. The Bessel beam, therefore, offers a higher degree of control for interactions at higher intensities^33^ in comparison to the Gaussian beam, with a high tolerance to positioning as well. These properties are related to the way the beam is formed, and the conical intersection of wavefronts requires typically a transparent environment. Given the successful use of Bessel beams for high aspect ratio structuring of transparent materials^39–47^, their approach on non-transparent/ metallic materials has been subject to discussion due to the required side geometry of waves interference for the beam formation. A non-transparent interface will intuitively arrest the beam formation process. However, a key advantage remains, i.e., the position tolerant irradiation in view of the beam length. This creates a considerable practical advantage to process surfaces with complicated shapes and topographies. At the same time, the question remains: how efficient is the ablation process with an obstructed beam and wavevector projections parallel and perpendicular to the surface of an opaque material? Several efforts have been made to demonstrate a certain feasibility of Bessel beams for ultrafast laser drilling of opaque materials, particularly metals and semiconductors. Kohno et al.^48^ and Matsuoka et al.^49^ reported the laser drilling by Bessel beams on steel plates with the thickness of up to 300 μm. The drilling by focused Gaussian beams was carried out at the same time, though no comparable evaluation was made between the Bessel and Gaussian beams. Alexeev et al.^50^ equally advocated the feasibility of Bessel beams for ultrafast laser drilling of metals assisted by a simplified time-invariant beam propagation model and ran an experimental verification of Bessel drilling on a copper foil. A concept of self-guiding was proposed from a numerical standpoint suggesting a more uniform propagation of Bessel beams and potentially higher efficiency.

Using a quantitative face-to-face comparison with Gauss drilling of stainless steel, we demonstrate here a two times higher drilling yield for a zeroth-order Bessel beam of similar fluence and waist. We identify the mechanism in the beam refolding on itself upon lateral reflections in the drilling channel, enhancing the core intensity and replicating the beam in the depth, well beyond its geometrical projection allowed by the entrance dimension. The preservation of the conical geometry allows for efficient self-healing around potential obstacles present in the path supporting in-depth energy delivery. While starting from similar surface ablation efficiencies, we unveil thus a super-efficient drilling process driven by the Bessel beam in the metal sample, where only the core is selected and sustained. The discussion approaches first superficial ablation i.e., low-depth ablation on stainless steel substrates under exposure to few pulses. In this low-depth ablation regime, the use of Bessel beams resulted in similar removal efficiency compared to the Gaussian counterpart. The result is reasonably verified with a two-temperature two-dimensional hydrodynamic model^51^, which suggests that the two beams initiate, expectedly, similar laser-matter interactions up to several microns depth. The discussion focuses then on deep drilling through a 200 μm-thick steel plate under exposure to thousands of pulses. In this case, for 200 μm-deep drilling, the Bessel beam demonstrates twofold higher efficiency than that obtained by the conventional Gaussian beam. Two keys aspects of the Bessel beam processing are considered to explain the observed effect: (i) Efficient self-propagation of the Bessel beam into the bulk material as evidenced from the topographic characterization of the laser-drilled holes in both top-view and cross-sectional profiles. (ii) Reduction of the plasma and particle shielding effects as suggested from a 3D propagation model. The Bessel beam self-replication and self-healing are considered for their contribution to overcome inherent particle shielding and scattering role and to advance in the bulk, ensuring efficient energy delivery beyond the surface.

## Results and discussion

### Analysis of Bessel versus Gaussian beam profiles

**Figure 1:**
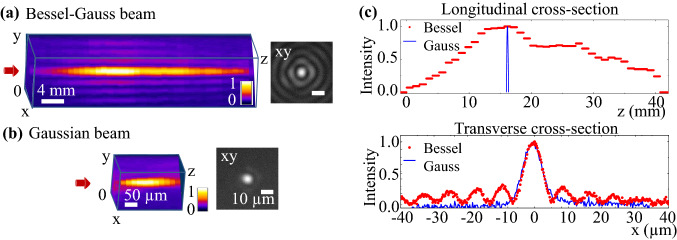
(**a**) Experimental analysis of the non-diffractive Bessel beam with the conical half-angle $\theta = 3^{\circ }$, the central core diameter $2r_{b}$ = 11 μm, and the Bessel length $z_{b}$ = 43 mm. The transverse beam profile consists of a high-intensity central core surrounded by a series of low-intensity concentric lobes. (**b**) Experimental analysis of Gaussian beam with the beam diameter $2r_{g} = 11$ μm at 1/$e^{2}$, and the depth of focus (confocal range) $z_{g}$ = 0.24 mm. The 3D illustration of the beams was stacked from transverse beam profiles individually captured along the propagation axis. (**c**) The on-axis cross-sectional profiles in the central core region of the beams (upper graph) and transverse cross-sections (lower graph).

In the present work, a Bessel-Gauss (BG) beam is generated by passing a collimated Gaussian beam through a glass (fused silica) axicon. The conical intersection of wavefronts upon propagation through the conical lens creates an interference pattern which is characterized as the zeroth-order Bessel beam that comprises an intense central core surrounded by a series of concentric lobes. The high-intensity central lobe does not spread within the non-diffractive propagation distance (Bessel zone) that can be evaluated as $z_{b} = D_{0}/(2\tan (\theta ))$, where $D_{0}$ is the diameter of the incident Gaussian beam. The Bessel beam was demagnified through a 4f telescopic afocal arrangement (demagnification factor 1.33).The schematic setup is described in the “Experimental and numerical methods” section. The resulting beam has a conical half-angle $\theta = 3$°, a diameter of central core $2r_{b}$ = 11 μm, and a Bessel length $z_{b}$ = 43 mm. The full width/diameter of the central lobe can be determined by the first zeros of the Bessel function^52,53^: $2r_{b} = 2\kappa /(k\sin (\theta ))$; where $k = 2\pi /\lambda$ is the wavenumber in air at the wavelength $\lambda$; and $\kappa \approx$ 2.405. The experimentally measured profile is shown in Fig. 1a. Here the 3D illustration of the beam was constructed from the transverse beam profiles, which were individually captured at different positions along the propagation axis *z*. The diameter of the central core was measured as the distance between the first zeros of the transverse Bessel beam cross-section. The contrast between the intensity of the first side-lobe and the peak intensity in the central core was estimated around 25%. The peak fluence at the central lobe can be approximated by^40^: $F_{b} = 4E_{in}/(r_{b}z_{b}\tan (\theta ))$; where $E_{in}$ is the Gaussian input energy. The fluence values were validated by the determination of the threshold values for material modifications, a property defined by the optical and thermomechanical characteristics of the material. The error range is approximately $10\%$.

For comparison, the conventional Gaussian beam was focused by means of a 4× objective with a focal length f = 45 mm and a numerical aperture NA = 0.1. The focal region is represented in Fig. 1b. The beam was characterized with a focused waist diameter $2r_{g}= 11$ μm, measured at 1/e$^{2}$, and a depth of focus (confocal zone) $z_{g}=0.24$ mm. Comparison of the on-axis intensity of the Gaussian and Bessel beams can be observed from the longitudinal cross-sections along the central core region (Fig. 1c upper graph). The confocal depth of the Gaussian beam is one order of magnitude shorter than the Bessel non-diffractive length. The transverse cross-sectional profiles (Fig. 1c lower graph) indicate a Gaussian beam diameter comparable with the central lobe of the presented Bessel beam. We note that for comparing the physical processes induced by the two beam types, the fluence is the relevant physical quantity from the energetic viewpoint, justifying the care for similar beam parameters.

### The efficiency of Bessel versus Gaussian beams for surface ablation

The ultrafast laser ablation on a stainless steel plate was evaluated with a series of static single-spot experiments using the Ti:Sapphire laser system at a pulse duration $\tau _L=100$ fs; the ultrashort range being more effective in terms of removal rate than longer pulses at similar fluence. For comparison, the Bessel-Gauss and the Gaussian configuration were evaluated at identical laser conditions, i.e., fluence, dose, spot dimension, and pulse duration. Throughout the experiments, each static exposure was delivered by a burst of 20 pulses; the number of pulses was chosen for creating measurable impacts (several μm depth) on the stainless steel sample. Under ablation by ultrafast laser Gaussian or Bessel beam, impact craters typically appear bearing a cylindrical shape. The ablated craters were characterized for their topographic profiles from which data on their depth and entrance diameter can be extracted.

**Figure 2:**
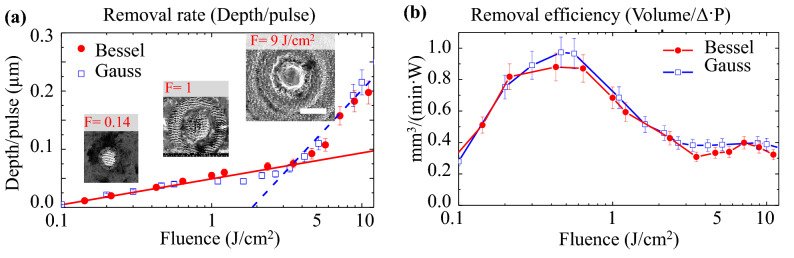
(**a**) Surface ablation for stainless steel, using ultrafast laser Bessel and Gaussian beams. The laser conditions were set identical at 1 kHz, 200 fs, and linear polarisation, while varying fluences. Removal rate curves show two different regimes: (i) Gentle regime (red solid-line) occurred at low pulse fluence ($<2~\text{J}/\text{cm}^{2}$), (ii) Strong regime (blue dash-line) occurred at high pulse fluence ($>2$ J/cm$^{2}$). Inset SEM images show topography of the craters ablated by the Bessel beam at different fluences (image scale: 10 μm). (**b**) Removal efficiency is evaluated by dividing the volume of the ablated crater produced in time unit by the power level. Both Bessel and Gaussian beams result in a similar efficiency trend with its peak within $0.5{-}1$ J/cm$^{2}$ fluence window. In the both cases, the local power density expresses the density of the energy rate through the surface in the interaction region and not the total power of the source.

For the evaluation of the removal rates, the maximum depth value of each ablated crater was normalized to the number of pulses, corresponding to the equivalent removal value for a single pulse (depth/pulse), and plotted as a function of the pulse fluence. As demonstrated in Fig. 2a, the use of the Bessel beam resulted in similar removal rates compared to the Gaussian configuration. The removal rate graphs indicate two ablation regimes that are typically observed in ultrashort laser machining on metals (typically below 1 ps): a gentle, shallow ablation regime with low ablation rates and a strong high rate regime, corresponding to respectively optical and thermal penetration regions^8,10^. These ranges are energetically separated by a fluence threshold around 2 J/cm$^{2}$. The inset scanning electron microscopy (SEM) images show the standard morphology of the craters ablated by the Bessel beam at different fluences.

In terms of ablation efficiency defined here as the removal yield most cost-effective in terms of photon cost, the measurement was defined by dividing the volume removal rate (ablated volume per time) per power unit: Volume/($\Delta t\cdot$P) in (mm$^{3}$/(min W)). The volume of each crater was calculated by multiplying the area of the circular entrance with its average depth. The ablation efficiency curves were plotted as a function of the pulse fluence. As demonstrated in Fig. 2b, both Bessel and Gaussian beams resulted in similar tendencies of ablation efficiency, with the peak of $0.8{-}1$ (mm$^{3}$/min W) corresponding to the fluence window of 0.5–1 J/cm$^{2}$. This range is accompanied by visible effects of excitation on the transient optical properties^54,55^. This peak efficiency window was in line with the numerical optimization model for Gaussian beams reported by Neuenschwander et al.^56,57^ that proposes a rule of thumb definition of the optimal fluence for efficient use of photons in the case of Gaussian beams $F_{opt}=e^{2}F_{th}$. In the particular case, the threshold fluence of steel is $F_{th}=0.1$ J/cm$^{2}$ in these irradiation conditions, which approximates the optimal fluence of $e^{2}$ × 0.1 $\approx$ 0.7 J/cm$^{2}$; experimentally in agreement with the fluence window of 0.5–1 J/cm$^{2}$. Here the maximum efficiency is around 1 mm$^{3}$/min W, which means that the ablated volume of 1 mm$^{3}$ is achieved in 1 minute using 1 W power unit. Further increase of the pulse fluence would lead to visible expulsion of molten material and re-deposition effects which deteriorate the ablation efficiency and quality. Based on the similar removal efficiency, the Bessel beam is suggested to initiate similar laser-matter interactions as the Gaussian counterpart at the micro-depth ablation scale, namely optical deposition of energy and rapid thermal transport with ballistic and diffusive components. The result is expectable as, in spite of a different beam formation geometry, the energy density on target is similar.

**Figure 3:**
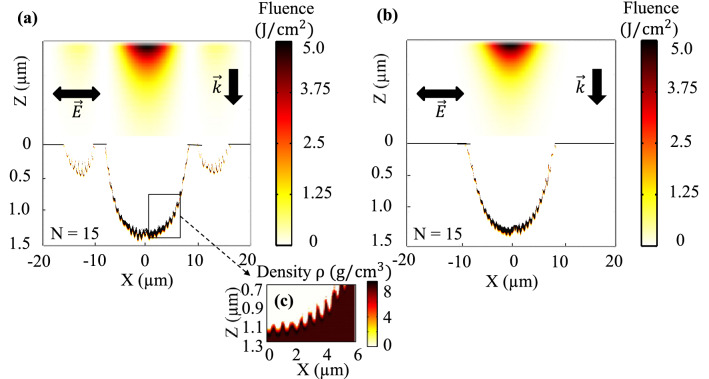
The simulated spatial profiles of the (**a**) Bessel-Gauss and (**b**) Gaussian beams of $2{r_{0}} = 11$ μm beam waist and peak fluence of $F = 5$ J/cm$^2$ are shown at the upper part of the figure. The energy penetration profile in terms of fluence and the corresponding morphology of the ablated craters after N = 15 pulse irradiation are provided at the lower part of the figure. Solid black lines identify the initial surface position at $Z = 0$. The density distribution of ripple morphology at the edges of the crater ablated by the Bessel beam taken after thermal equilibrium at 10 ps is magnified in (**c**), indicating the amount of the ablated material (red color) at densities below the liquid state $< 6.9$ g/cm$^3$. The average ablation rate $\approx 90{-}100$ nm/pulse.

Numerical simulations were performed to compare the ablation efficiency of ordinary Gaussian and Bessel beams in the surface ablation regime down to a depth of few microns. The ablation model developed in Ref.^51^ solves self-consistently electromagnetic, thermal, and hydrodynamic equations and couples light distribution, absorption, and material movement, being able to consider at the same time the light action on the matter and the feedback action of modified matter and topography on the light. The model was applied to evaluate the action of $N = 15$ pulses at a fixed fluence of $F_{b} = 5$ J/cm$^2$. The results shown in Fig. 3a,b, namely the achieved depth of 1.5 μm, agree with the average ablation rate of $\approx 90{-}100$ nm/pulse measured experimentally. They equally put into evidence a complex morphology of the ablated craters (blow-up given in Fig. 3c), with near-wavelength, 550 nm ripple structures at the edges of the crater (as experimentally observed in Fig. 2a, resulting from coherent scattering and generation of surface waves that lead to a standing pattern modulated in a first degree with a near-wavelength period^58,59^. For a Bessel beam, the ablation takes place not only by the central lobe but also by the high order side-lobes, resulting in smaller impacts on a distance of $\approx 15$ μm from the center, visible in Fig. 3a. The ablation depth of the central crater appears to be comparable in both Bessel and Gauss cases.

### The efficiency of Bessel versus Gaussian beams in deep drilling

In the deep drilling regime, the Bessel beam is evaluated for its efficiency by drilling through a 200 μm steel plate and comparing the result with the Gaussian counterpart. The results are evaluated in terms of the time required for crossing the plate or of the channel depth in the material. So as to determine the required through-drilling time, the experiments deploy photodiodes for detecting the pulse signals exiting from the drilled holes. The time taken to drill through was recorded and converted to the total number of pulses considering the laser repetition rate. Both Bessel and Gaussian beams were used with the same configurations as described in the previous section. The laser conditions were kept identical throughout the experiments.

**Figure 4:**
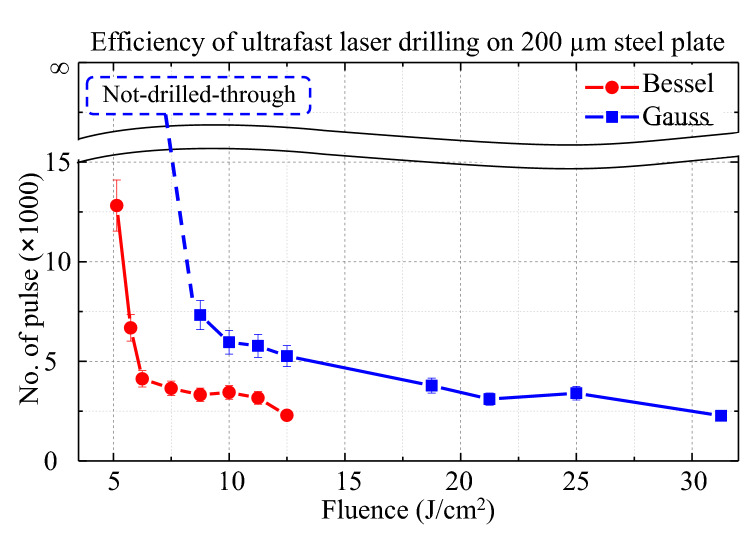
Efficiency comparison of the ultrafast laser drilling through the 200 μm thick steel plate in the Bessel and Gauss modes. The results are presented as the overall number of pulses necessary to pierce through the plate as a function of the pulse fluence (*F*). A smaller number of pulses is generally required to drill through the plate upon increasing the laser fluence. The use of the Bessel beam resulted in a twofold drop of the required pulse number (red-curve) when comparing to the Gaussian beam (blue-curve) in the tested fluence range. Particularly, at the fluence below 8 J/cm$^{2}$ the Bessel beam was still able to drill through the plate while the Gaussian resulted in incomplete, blind channels (represented by an infinite number of pulses).

Represented in Fig. 4, the results of the laser percussion drilling were plotted as the number of pulses required to pierce through the plate as a function of the pulse fluence (effective drilling-through dose). Generally, the curves show a descending trend of the required pulse numbers as the fluence increases. This descending trend can be expected since the increase of fluence induces the removal of larger amounts of ablated material, which shortens the required time or, alternatively, the pulse numbers necessary to drill through the plate. However, the comparison between the two beam configurations shows that the Bessel beam through-hole drilling required, on average, a two-times smaller number of pulses (Fig. 4, red-curves/bars) than the Gaussian beam (Fig. 4, blue-curves/bars), at the same fluences in the tested drilling-through region. Particularly, at a fluence below 8 J/cm$^{2}$, the Bessel beam was able to drill through the plate, whereas the Gaussian beam drilling resulted in not-drilled-through/ blind holes. One can argue that the Bessel beam drills on average twice efficiently as the Gaussian beam. Concerning the potential effects of laser polarisation, the difference was negligible in both cases of Bessel and Gaussian beams. The pulse numbers seem to experience only a small change when switching from linearly polarised beams to circularly polarised beams (data not shown due to their redundancy). Given their similar ablation rates at the low-depth ablation scale, the higher drilling efficiency of the Bessel beam over the Gaussian beam is rather unexpected and requires an approach that integrates their particular propagation to uncover the underlying physics.

#### Propagation of the beams into the bulk material

Given the way they are formed via conical intersection, the Bessel beams are by nature quasi-stationary patterns, requiring energy delivery and photon replenishment from the sides. An apparent propagation effect relies on the gradual intersection of the wavefronts as the axicon-refracted beams progress and it corresponds to different regions within the radius of the incident beam being concatenated on the axis. An opaque material where the interface arrests the conical wave propagation (with the wavevector making an angle $\theta$ with the axis) should impede their axial overlap and interference, and as such, the formation of the beam beyond the surface. Nevertheless, with an opening of the crater, they penetrate with extreme efficiency in the corresponding channel space. Their geometrical propagation represents the projection of the entrance along the axis, with a propagation length $L_{bc}=R_c/tan(\theta )$, with $R_c$ being the radius of the channel opening. Axial projections $L_{bc} = 100$ μm are obtained for a crater diameter in the 10 μm range. This represents a unique property of the advance of the Bessel beam into the bulk stainless steel material during the drilling process. Notwithstanding this observation, the Gauss beam represents a propagative wavepacket with a comparable Rayleigh range of 120 μm.

**Figure 5:**
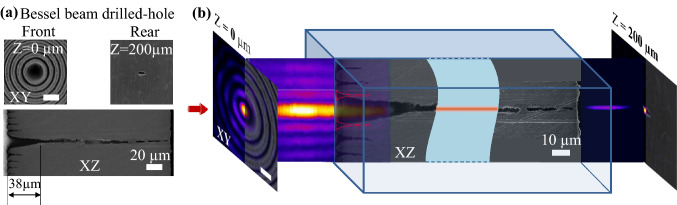
(**a**) SEM images of the hole drilled through the 200 μm steel plate by the Bessel beam at the laser fluence $F= 5.15$ J/cm$^{2}$. Upper images show top-view surfaces of the hole front whose topography resembles the arrangement of the concentric lobes of the Bessel beam profile, with an entrance ($Z=0$) hole diameter of $\sim 24$ μm and the hole rear ($Z=200$), with an average diameter of $\sim 7$ μm. The lower image displays the cross-sectional profile of the hole along the longitudinal axis *Z*, with a tapering distance of $\sim 38$ μm. (**b**) Matching of the Bessel beam profiles to the surface topography of the drilled hole. Ablation occurred at not only the 0th-order central lobe but also the 1st-order side lobe. Higher-order side lobes are equally leaving isolated traces. Imaginary drawing (the red-dot line region) was supposed for the ablated shape of the 1st-order side-lobe. However, the actual tapering shape indicates a bend of the 1st-order side-lobe toward the center. A replicated version of the Bessel beam is suggested to be formed and drills through the plate. The replicated Bessel beam profile was captured after exiting the drilling-through hole.

In order to trace evidence of the beam propagation features, we exposed the axial sections of the drilled holes, following a series of molding and polishing processes and scanning electron microscope characterization (see the “Experimental and numerical methods” section). For the Bessel case, Fig. 5a shows top-view (upper images) and longitudinal (lower image) cross-sections of a particular hole drilled by Bessel beam at the peak fluence $F_{b}$ = 5.15 J/cm$^{2}$. The front surface of the hole bears the fingerprint of the Bessel beam profile with the hole entrance diameter of $\sim 24$ μm, surrounded by concentric ablated lobes. The rear surface appears as a single opening with an average diameter of $\sim 7$ μm. It is worth noting that the front surface profile and hole entrance diameter remain relatively constant regardless of varying ablation fluences, a consequence of a Bessel profile with the rapid decay of fluence and the zeroing points. In depth, the longitudinal cross-section of the drilled-through hole reveals a tapering distance of $\sim 38$ μm which accounts for $\sim 20$% of the plate thickness. The through-hole exceeds the geometrical projection of the beam, suggesting a mechanisms of light propagation within the channel that is sustained over a long distance. The ring penetration, given the typical contrast of a Bessel-Gauss beam, gives a first impression of the drilling efficiency as a function of the fluence.

From matching of the Bessel beam profiles to the surface topographies of the drilled holes (Fig. 5b), it can be seen that the entrance hole was created by the ablation at not only the zeroth-order central lobe location but also involved the 1st-order side-lobe. Supposedly the ablation at the 1st-order side-lobe occurred similarly to the higher-order side-lobes, albeit the fluence difference, the shape of the removed material could be imaginarily drawn up as the red-dot line region. However, the actual tapering shape indicates a bend of the 1st-order side-lobe toward the center. This is the consequence of an apparent shift of the lobe due to a change of path of the interfering beams upon reflection on the crater. This is a first element suggesting that the beam folding and replication start to take place in the cone. The presence of metal particles in the channel can equally determine a change in the effective refractive index during propagation. The process continues up to the point where the tapered entrance filters the main lobe that continues to propagate quasi-independently without being sustained by the side lobes reservoir. A similar effect occurs for the higher-order rings. Each component initially formed by intersection and interference will autonomously propagate, and the individual imprints induced by the high-order Bessel lobes gradually move from interference patterns to trapped light that propagates in the side-ablated craters. If the side lobes are rapidly damped by the unfavorable geometry, the main lobe, filtered out from the pattern, continues its propagation. The specific orientations of the wavevectors at the $\theta$ angle with respect to the axis suggest a replication by lateral reflection. The as-propagated Bessel beam can be observed after exiting the drilled-through hole (Fig. 5b lower-right).

**Figure 6:**
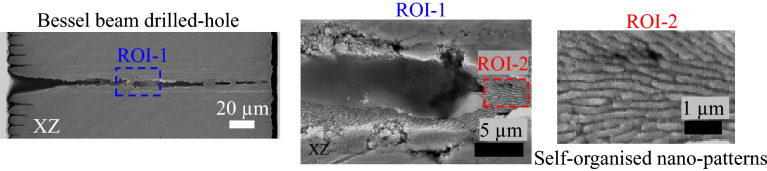
SEM cross-sectional image of the hole drilled through the 200 μm steel plate by the Bessel beam at the laser fluence F = 5.15 J/cm$^{2}$ (Left); Blow-up images in the ROI-1 (Middle) and ROI-2 (Right), showing the self-organized nano-structures induced by the self-replication of the Bessel beam.

The argument of the self-replicated Bessel propagation via reflections is additionally supported by a second argument; the topography of the drilled hole walls. The observation of self-organized periodic nanostructures on the channel walls (Fig. 6) with a rather constant periodicity in the 180–200 nm range indicates the correspondence to high spatial frequency ripples. These nanoscale patterns show thus a direct incidence of the laser beam on the channel walls triggering self-organization through light scattering and thermal transport of a rough surface^51,60^.

A further aspect becomes apparent in the self-replication process. It is worth mentioning that some segments of the channel were observed with widths below 7 μm, which is much narrower than the central core diameter of the original Bessel beam, possibly induced by a crater-driven modification of the cone angle.

**Figure 7:**
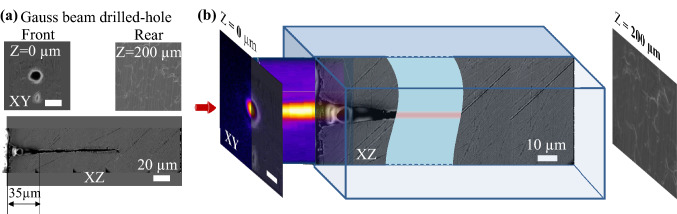
(**a**) SEM images of the blind hole drilled in (not through) the 200 μm plate by the Gaussian beam at the laser fluence $F_{g}$ = 6.25 J/cm$^{2}$. Upper images show top-view surfaces of the hole front ($Z = 0$) whose topography resembles the Gaussian beam profile, and the rear ($Z = 200$ μm) indicating the blind end. Lower image displays cross-sectional profile of the hole along the longitudinal axis *Z*. The blind hole has a tapering distance $Z_{tap}\sim 35$ μm and a drilled depth of 135 μm. (**b**) Matching of the Gaussian beam profiles to the surface topography of the drilled hole. The hole entrance diameter of 13 μm is larger than the Gaussian beam diameter 11 μm.

For comparison, in the case of drilling at a similar fluence range, i.e., smaller than 8 J/cm$^{2}$, the Gaussian beam resulted in not-through blind holes. Figure  7 shows profiles of a blind hole drilled by the Gaussian beam at the particular fluence $F_{g}$ = 6.25 J/cm$^{2}$, which is already larger than the fluence $F_{b}$ = 5.15 J/cm$^{2}$ used for the drilled-through hole by the Bessel beam. The Gaussian-drilled blind hole was observed with a depth of 135 μm which is $\sim 67$% of the plate thickness. At this fluence, the depth is similar to the Rayleigh range. Sidewall reflections^61^ can equally sustain further propagation of the Gauss beam; however, in view of the angular dispersion of the beam (convergence angle $\theta _{G}: 5.7^{\circ }$), the effect seems significantly reduced. The tapering profile also occurred within $\sim 35$ μm distance. From matching of the Gaussian beam profiles to the surface topography around the drilled hole, the hole entrance can be seen slightly larger (13 μm) than the Gaussian beam diameter (11 μm) suggesting fluences exceeding the threshold of spots wider than the 1/$e^{2}$ beam width.

#### Plasma and particle shielding effects

The ultrafast laser ablation of metals involves a complex sequence of physical processes and high removal rates are usually associated by the ejection of liquid nano- and micro-particles. This is inherent to thermal trajectories in the vicinity of the binodal or in the mixed-phase region^62^. In case of deep drilling, these heavy plume components cannot be swiftly evacuated from the channel and create screening and shielding effects, partially absorbing and scattering the incident laser energy^63,64^. The consequence is direct deterioration of the ablation rate and efficiency. In this case the reconstruction capability of the central lobe of the Bessel beam can be more efficient to overcome the limitation and to ensure a higher drilling efficiency, transporting the energy deeper in the depth. However, if we estimate the effect to be reasonably reduced given the 1 kHz repetition rate of the present experiment (similar efficiency was noted for a repetition rate varying between 10 and 1000 Hz), a long-living presence of particulates may be assumed.

#### Numerical insights into beam propagation

**Figure 8:**
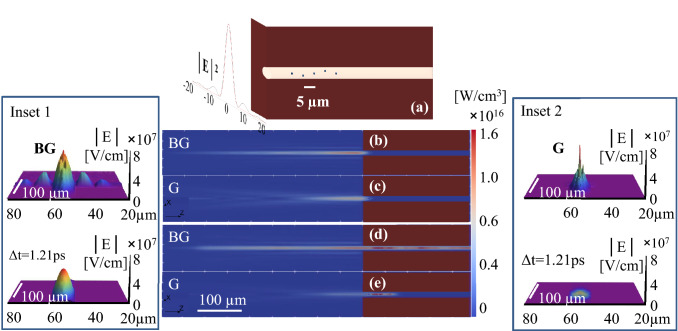
Beam traces (accumulated power density of the traveling wave) during the interaction with the metal target for two particular moments in the simulation. (**a**) The schematic description of the modeling geometry. (**b**) Energy density $\rho _P$ for the Bessel beam before the beams arrive the focus on the metal surface. (**c**) $\rho _P$ for the Gauss beam before the beams arrive at the focus. (**d**) $\rho _P$ for the Bessel beam before the end of the channel. (**e**) $\rho _P$ for the Gauss beam before the end of the channel. The Bessel beam penetrates far deeper into the channel replicating successively in space the beam projection from the entrance. The insets show the instantaneous electric field (modulus) distributions of the Bessel (Inset 1) and the Gauss (Inset 2) wavepackets at the entrance of the crater and in the crater end (sectional view), with a time delay difference of 1.21 ps. One notes the field enhancement at the entrance and the field reshaping within the structure, as well as the efficient damping of the Gauss beam.

An appropriate propagation model that couples the spatial and temporal evolution of the ultrashort laser pulse is needed for further examination of the physical mechanisms behind the different drilling dynamics between the Bessel and Gaussian beams. To this end, we have modeled Gaussian and Bessel beam sources and evolution in free space and in confined environments using a finite difference time domain formalism (see the “Experimental and numerical methods” section). The simulation includes the formation of the source via an axicon of $D_a=$ 100 μm diameter and base angle $\alpha =5^{\circ }30^{\prime}$, giving a half-cone angle of $\theta _b=2^{\circ }30^{\prime}$ (roughly equivalent to the virtual axicon projected by the 4f experimental imaging setup) or via a lens of an equivalent numerical aperture, exposed to an incident Gauss beam of a diameter $D_g=$ 45.6 μm. The focusing optical elements are chosen to develop radiation sources keeping approximately the same diameter of the Bessel and the Gauss beams ($r_b=6.8$ μm Bessel radius at the first zero and $r_b=5.35$ μm Gauss waist radius at $1/e^2$). Thus the Gauss envelope approximates the envelope of the central lobe of the Bessel beam. Due to considerations of simulation time, the numerical source was downsized with respect to the experimental situation to a $z_b=0.55$ mm ($z_b=D_g/2\tan (\theta _b)$), capturing nevertheless the essential elements of beam evolution. In these conditions, a 100 fs Gaussian wavepacket incident on the axicon has a coherence length $\tau _L\cdot c = 30$ μm (*c* being the speed of light in vacuum), on the same order of magnitude with respect to the lateral extent of the refracted wave (22.8 μm). The consequence is that the non-diffractive length of 0.55 mm is constructed progressively by interference slots defined by the coherence length (axial projection $l_c/\cos {\theta }$ = 30 μm) when the refracted wavepackets cross, sweeping the longitudinal direction. The two beams deliver the same power ratio per unit area in the focus region where we place the target. The metal target contains a void channel of $D_{ch}=12$ μm diameter and both beams propagate in the hollow structure. The expected Bessel projection through the entrance is 139 μm, to be compared to a characteristic Rayleigh range of the Gauss beam of 85 μm. The channel contains a number (5) of steel particles of cubic shape and 200 nm size arranged at equidistance close to the entrance, within a range of 25 μm, as shown in Fig. 8a. The simulation accounts thus for the propagation in the channel while encountering nm-scaled obstacles that scatter light and define an effective index for the propagation environment. We then track the power density trace in time of the traveling wave $\rho _P$ representing the energy flow and delivering the fingerprint of the beams. Figure 8 (Inset 1,2) sketches two relevant moments in the simulation. Figure 8b,c illustrates the radiation trace (power density history) $\rho _P$ along the Z-axis as the beams arrive the focus for Bessel-Gauss (BG) and Gauss (G) beams, respectively. Figure 8d,e traces the complete history of the beams as they reach deep into the channel. The insets deliver the instantaneous electric field (absolute value) of the Bessel and Gauss wavepackets at the entrance of the crater and at the end of the crater (time difference 1.21 ps).

We observe that the entrance of the channel filters out the higher lobes for the Bessel beam limiting the conical delivery of energy through the opening only. The channel diameter comparable with the hole diameter truncates both beams and generates diffraction. This first distortion element triggers a relatively important field enhancement as both beams enter the channel (Fig. 8a,b). However, along the channel the situation is different. We observe first that the Bessel projection through the channel entrance has a similar characteristic dimension as the Rayleigh range, i.e., around 100 μm. In a first approximation one should then expect a similar penetration depth. The modeling result shows a significantly higher advance for the Bessel beam, almost three times deeper within the limits of the channel. First, the beam trace is spatially modulated, recreating the projection through the channel at different penetration depths. This indicates a propagation appearance which is sustained by the boundary conditions through repeated reflections from the channel metallic walls. The lack of angular dispersion of the Bessel beam with a defined direction of the wavevectors favors this propagation mode (Fig. 8c). The electric field is reshaped in the channel (see inset) as the wavepacket advances through a succession of divergence and convergence events. The channel-supported propagation is less efficient for the Gauss beam due to a higher angular dispersion of the wavevectors, affecting the reflectivity from the walls at quasi grazing incidence, thus with a cost in uniformity and in the energy propagation of several percent. However, the dominant effect comes from the presence of the nanoscaled scatterers. They efficiently damp the Gauss beam within its Rayleigh range; with the process being effective even at a low density of particles (5 particles in our case). This damping effect is related to a highly efficient scattering processes enabled by the nano-scatterers, e.g. Mie scattering. The presence of rough channel walls may produce a similar effect. The conical geometry of the Bessel beam instead allows the reconstruction of the beam around the obstacles ensuring thus its endurance. Altogether, a Bessel-specific beam advance in a channel permits to transport energy on far larger distances than the Gauss beam, with self-healing and regeneration being an efficiency booster for the drilling process.

## Conclusion

We report a super-efficient drilling mechanism enabled by ultrafast non-diffractive beams in opaque materials. The drilling rate through a 200 μm-thick steel plate of the zeroth-order Bessel beam is found twice as high as an equivalent Gauss beam for moderate fluences, albeit a similar efficiency for surface ablation processes confirmed by hydrodynamic modeling. The mechanism sustaining a more effective propagation of the Bessel beam relies on the self-replication and the reconstruction of the Bessel beam main lobe into the drilling channel in the presence of nanoscale scatterers. FDTD simulation uncovers a dynamic interplay of reflections facilitated by the conical geometry of wave-vectors and self-healing around scattering particulates, replicating and enhancing the beam on the propagation axis. A highly efficient and unique drilling process is underlined. The results show the key importance of the beam design, ensuring for the same energetic conditions stronger yield and throughput of advanced laser processing methods.

## Experimental and numerical methods

### Bessel and Gauss irradiation setup

**Figure 9:**
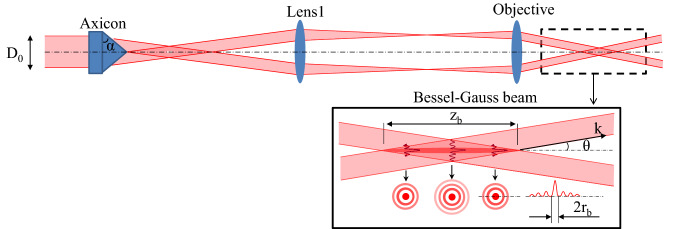
Schematic of the Bessel-Gaussian beam configuration, including an axicon and a standard 4f telescopic system (an achromatic lens and a microscope objective). The inset describes standard parameters of the Bessel beam with $D_0$ is the incident Gaussian beam diameter, $\alpha$ is the physical or base angle of the axicon, $Z_b$ is the Bessel length, $\theta$ is the half cone angle, k is the wave vector, and $2r_b$ is the diameter of the beam central core.

The irradiation unit is based on a Ti:Sapphire amplified laser system at an operating repetition rate of 1 kHz (equipped with a pulse selector), central wavelength $\lambda _L=800$ nm and pulse duration $\tau _L$ adjustable between 100 fs and 10 ps. Mostly the short pulse was used. The setup includes an axicon versus a standard achromatic lens and a 4f telescopic imaging system projecting the beam on the metal surface, with a microscope objective as the final element (Fig. 9). The specific irradiation parameters are given in the text. The metal plate is scanned using a three-axis positioning system.

### Impact characterization

Impact visualization in deep drilling is achieved using scanning electron microscopy in transverse and axial sections after molding and polishing. In the shallow ablation range, the depth is measured using confocal microscopy. Threshold measurements were performed by the dimensional analysis of the craters.

### Electromagnetic simulation of beam propagation

Beam propagation is an essential process assisting drilling and we developed an electromagnetic approach to follow far-field beam evolution starting from the source. A vectorial Finite Difference Time Domain (FDTD) formalism is used for the geometry given in the text, following the propagation of various beams in a metallic hollow channel containing nanoparticles. We define the power that each Yee’s cell^65^ contains by using the expression^66^ $I_{cell}(\mathbf {r},t)=\iiint _{cell}\left( \mathbf {E}(\mathbf {r},t)\cdot \nabla \wedge \mathbf {H}(\mathbf {r},t) - \mathbf {H}(\mathbf {r},t)\cdot \nabla \wedge \mathbf {E}(\mathbf {r},t)\right) dV$, allowing to define the energy flow and equally, the accumulated energy per cell. In the interval of time $\Delta _t'=\frac{\Delta _{z}\sqrt{\varepsilon _r\mu _r}}{c_0}$, the beams run a cell length in the Z-axis. The energy flow depicts thus the trace of the beam as $\rho _P=|\triangledown \cdot \mathbf {S}|$, with *S* being the Poynting vector. The time integration provides the accumulated energy per cell $E_\Sigma (\mathbf {r})=\int _{0}^{t_{end}}\Delta _t'\left( I_{cell}(\mathbf {r},\tau )\right) ^2d\tau$, where $t_{end}$ is the simulation duration. From a numerical point of view, this integration is carried out by means of a sum $E_\Sigma (\mathbf {r})\simeq \Delta _t\Delta _t'\sum \limits _{n=0}^{N_t}\left( I|^n_{i,j,k}\right) ^2$ where $t_{end}=\Delta _t N_t$ being $\Delta _t$ the time-stepping size for the algorithm updating and $N_t$ the number of steps. The power per unit of cell is given by $I_\Sigma (\mathbf {r})=\frac{E_\Sigma (\mathbf {r})}{\Delta _t\Delta _t'}=\sum \limits _{n=0}^{N_t}\left( I|^n_{i,j,k}\right) ^2$. The metal target complex refractive index^55^ is $\tilde{n}=2.57+i3.12$ where optical properties at relatively high electronic temperatures (30,000 K) were used to emulate laser-material interaction and the excitation of the metal. This electronic temperature marks the beginning of material destructuring^67^ and is regarded as a reasonable hypothesis for interaction with hot but not ablated walls.

### Electromagnetic-hydrodynamic ablation model and multipulse feedback

The approach describes the close range interaction with the material. A surface interaction model is used to estimate the superficial ablation process starting from an incoming distribution of energy coupled to the material. The electromagnetic-hydrodynamic ablation model was reported in Ref.^51^ and couples self-consistently light and material effects, notably the feedback of topography on the light patterns at the surface. At each pulse, accounting for scattering and surface interference effects, the absorbed energy was calculated by solving Maxwell equations with excitation-dependent material parameters^55^, then the two-temperature model and Navier-Stokes equations were solved in order to get material temperature and density distributions, providing the onset of phase transitions and the amount of the ablated material with the resulting crater topography. The processes of plasma plume expansion in air, material redeposition and fluid movement occurring on a long time scales after electron-ion thermal equilibrium were not considered here due to the complexity of processes at high ablation fluence. The model allows for multipulse simulations for hole drilling by Gaussian and Bessel-Gaussian beams. To simulate a focused Bessel-Gaussian beam profile, the initial electric field source is introduced at $z=z_0$ as follows.

1 $$\begin{aligned}{E_x}(t, r, z_0) & = \frac{w_0}{w(z_0)}{\exp }\left[ (r^2+\frac{\beta ^2{z_0}^2}{k^2})(-\frac{1}{w^2(z_0)}\right. \nonumber \\ & \quad \left. +\frac{ik}{2R(z_0)}) + i(k-\frac{\beta ^2}{2k})z_0 - i\varsigma (z_0)\right] \times {J_0}\left( \frac{\beta {r}}{1+iz_0/Z(r)}\right) \nonumber \\&\quad {\exp }\left[ -\frac{(t-t_0)^2}{\tau ^2}\right] . \end{aligned}$$

where $r = x^2 + y^2$, *w*(*z*) is the beam width, $\varsigma (z)$ is the phase-shift, $R(z) = z\left[ 1+(Z(r)/z)^2\right]$ is the radius of curvature and $Z(r) = \pi {w_0}^2/\lambda$ is the Rayleigh range, defined analogically to ordinary Gaussian beam with beam waist of $w_0 = 5.5$ μm and central wavelength $\lambda = 0.8$ μm, $\beta = k{\sin }(\theta )$, $\theta = 3^{\circ}$ is the inclination angle with respect to the propagation axis *Z* or conical half-angle, and $J_0(r) = \frac{1}{2\pi }\int _0^{2\pi }\left[ {\exp }(ir{\cos }\alpha )d\alpha \right]$ is the cylindrical zeroth-order Bessel function^68^, $\tau$ is the pulse duration, and $t_0$ is the time delay. The Bessel function is approximated by a finite sum of $N = 20$ finite waves. In a particular case of $\beta = 0$, the profile represents an ordinary focused Gaussian beam. The peak fluence is defined as $F = \frac{\tau }{2}\sqrt{\frac{\varepsilon _0}{\mu _0}}\left| \mathbf {E}\right| ^2$, where $\varepsilon _0$ and $\mu _0$ are the vacuum permittivity and permeability.
